# Masson's Tumor of the Hand: An Uncommon Histopathological Entity

**DOI:** 10.1155/2020/4348629

**Published:** 2020-03-18

**Authors:** Parviz Mardani, Amir Askari, Reza Shahriarirad, Keivan Ranjbar, Amirhossein Erfani, Mohammad Hossein Anbardar, Shirin Moradmand

**Affiliations:** ^1^Thoracic and Vascular Surgery Research Center, Shiraz University of Medical Science, Shiraz, Iran; ^2^Department of Surgery, Shiraz University of Medical Sciences, Shiraz, Iran; ^3^Student Research Committee, Shiraz University of Medical Sciences, Shiraz, Iran; ^4^Department of Pathology School of Medicine, Shiraz University of Medical Sciences, Shiraz, Iran

## Abstract

Masson's tumor or Masson's hemangioma, more precisely termed intravascular papillary endothelial hyperplasia (IPEH), is an uncommon benign vascular lesion of the skin and subcutaneous tissues which can be frequently confused with angiosarcoma. Although relatively rare, its accurate diagnosis is essential since it can clinically be similar to both benign and malignant lesions. We present a 39-year-old man with a round bulging arising from the left palm side of the hand with gradual growth in the last 5 months and on and off tenderness. The microscopic section demonstrated the papillary proliferation of endothelial cells in favor of Masson's hemangioma, which was sufficiently treated with excision alone.

## 1. Introduction

Intravascular papillary endothelial hyperplasia (IPEH) was first described by Pierre Masson as an intravascular papillary proliferation that forms within the lumen of an inflamed hemorrhoidal plexus, which was named vegetated intravascular hemangioendothelioma. Since then, various terms were considered for this pathology, such as hemangioendothelioma and reactive papillary endothelial hyperplasia or better known as Masson's hemangioma [1–3]. It can arise from normal blood vessels or in vascular malformations. The precise pathogenesis of the disease is still unclear, although the response to blood vessel injury or thrombosis may be accountable for this situation [4, 5]. IPEH is an uncommon benign vascular lesion of the skin and subcutaneous tissues [6]. When occurring on the hand, it may be difficult to differentiate from other soft tissue lesions of the extremity by clinical examination alone. While the prognosis is excellent, differentiation from other causes that require more extensive interventions, such as angiosarcoma, is essential [7].

## 2. Case Report

A 39-year-old man with a complaint of a round mass on the palmar side of his left hand was referred to our clinic. The lesion had on and off tenderness and was gradually growing in size since 5 months ago. In our physical exam, there was a 4 × 3 cm, firm, round, and nontender lesion on the palmar side of the left hand over the second metacarpophalangeal joint, without a change in skin color or dense adhesions to surrounding soft tissues.

With the impression of a benign hand lesion, without any further evaluation, the patient was scheduled for excisional surgery. Under local anesthesia, via a vertical incision over the lesion, the mass was enucleated easily, the wound was closed without any complications, and the lesion was sent for pathological evaluations.

**Figure 1:**
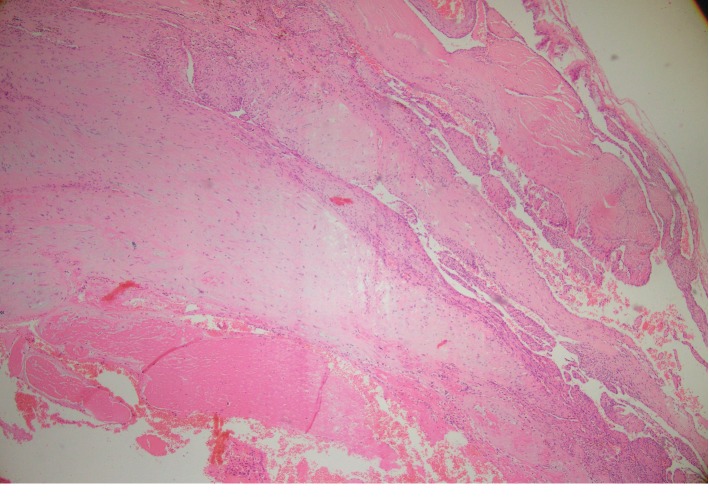
Microscopic section shows a vessel wall with papillary proliferation of endothelial cells and thrombus formation at the center (hematoxylin and eosin, ×40).

**Figure 2:**
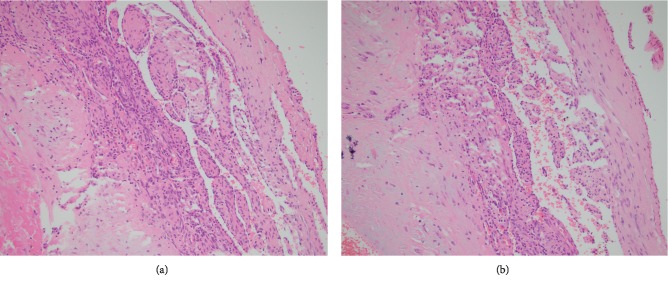
(a, b) Microscopic section shows papillary proliferation of endothelial cells (hematoxylin and eosin, ×200).

**Figure 3:**
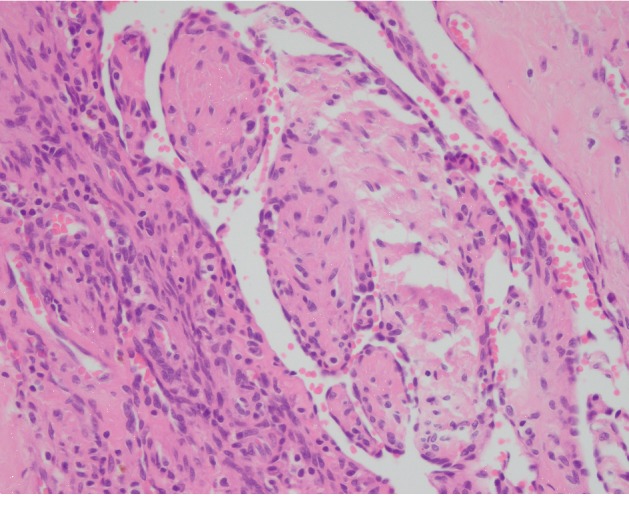
Microscopic section shows a high-power view of papillary proliferation of endothelial cells without any atypia or hyperchromasia (hematoxylin and eosin, ×400).

The gross examination of the lesion revealed an ovaloid gray-brown color cystic mass measuring 3.5 × 2 × 2 cm, in which the cut section revealed cystic mass filled with clot material. Microscopic examination showed dilated vessels containing papillary proliferation of plump endothelial cells without atypia overlying fibrous tissue (Figures 1, 2(a) and 2(b), and 3). Also, there was thrombus formation and fibrin deposition. No evidence of mitosis or necrosis was observed. The patient had a good surgical outcome and required no further surgical intervention.

## 3. Discussion

IPEH constitutes about 2-4% of the benign and malignant vascular tumors of the skin and subcutaneous tissues [8]. There is no age predilection and it has been equally reported in males and females. However, Some studies have reported a female preponderance [9]. Pins et al. described in their study a female to male ratio of 1.2 : 1.0 with an average age presentation of 34 years [10]. IPEH usually presents as a small, firm, superficial mass with slow growth [11, 12]. It may occur in any part of the body but the most common locations are hands, fingers, head, and neck, respectively [11]; however, oral mucosa, lip, thyroid, maxillary sinus, parotid, lung, superior vena cava, adrenal gland, renal vein, forearm, foot, and the intracranium have also been reported [1, 13–17].

As illustrated in our study, this entity is characterized by multiple small papillary proliferation of endothelial cells and thrombus formation at the center. The exact pathogenesis of IPEH is unknown, but an unusual organization of the thrombus after trauma is considered to play a key role [4, 18, 19]. After a mechanical stimulus to vessels, the release of fibroblast growth factor (FGF) by migrating macrophages to the injured site can cause the proliferation of endothelial cells. This proliferation can amplify more FGF release and produce positive feedback on endothelial cell proliferation [20].

The main method for diagnosis is histological evaluation; however, IPEH can histologically simulate angiosarcoma, which makes distinguishing these two entities an important issue to avoid aggressive resection. Angiosarcomas are almost never confined to the vascular lumen; thus, the intravascular location of IPEH may assist in confirming the diagnosis. However, although rare, the extravascular subtype may result in a misdiagnosis. The differentials should also include pyogenic granuloma, Kaposi sarcoma, and other vascular conditions [7].  Table 1 demonstrates a number of differential diagnosis which should be considered for Masson hemangioma, along with their pathological features.

Simple excision is usually curative, although recurrence has been described [21]. It is essential to differentiate IPEH from angiosarcoma to avoid unnecessary surgery and radiation [17]. Treatment of the lesion consists of conservative surgical excision with good outcomes in all cases except intracranial lesions, which have been reported to be fatal [22].

## 4. Conclusion

In conclusion, Masson's hemangioma can be misdiagnosed as a benign lesion as well as malignant neoplasm due to its clinical resemblance. Therefore, it is important to distinguish IPEH from other benign and malignant lesions, in particular, angiosarcoma to avoid unnecessary radiation and surgery. It can also be misdiagnosed as other conditions, such as a tendon cyst, on ultrasound evaluation. For these reasons, accurate diagnosis can solitarily be completed through histological examination.

## Figures and Tables

**Table 1:** Differential diagnosis of Masson's hemangioma and pathological features.

Diagnosis	Cellular pleomorphism	Solid areas and necrosis	Papillary architecture	Intravascular location	Piling of endothelial cells
Masson's hemangioma	Rare	No	Common	Common	Rare
Cavernous/capillary hemangioma	Rare	No	No	No	Rare
Kaposi sarcoma	Common	Uncommon	No	Rare	Rare
Endovascular papillary angioendothelioma	Common	Rare	Common	Common	May be present
Angiosarcoma	Common	Common	Rare	Rare	Common
